# Cigarette Smoke Affects ABCAl Expression via Liver X Receptor Nuclear Translocation in Human Keratinocytes

**DOI:** 10.3390/ijms11093375

**Published:** 2010-09-17

**Authors:** Claudia Sticozzi, Alessandra Pecorelli, Giuseppe Belmonte, Giuseppe Valacchi

**Affiliations:** 1 Department of Biomedical Sciences, University of Siena, Via Aldo Moro, 2 53100 Siena, Italy; E-Mails: sticozzi@unisi.it (C.S.); belmonte4@unisi.it (G.B.); 2 Department of Physiopathology, Experimental Medicine and Public Health, University of Siena, Via Aldo Moro, 2 53100 Siena, Italy; E-Mail: pecorelli@unisi.it; 3 Department of Food and Nutrition, Kyung Hee University, 1 Hoegi-Dong, Dongdaemun-gu–Seoul, Korea

**Keywords:** ABCA1, keratinocyte, LXR, cigarette smoke, NFκB

## Abstract

Cutaneous tissue is the first barrier against outdoor insults. The outer most layer of the skin, the stratum corneum (SC), is formed by corneocytes embedded in a lipid matrix (cholesterol, ceramide and fatty acids). Therefore, the regulation of lipids and, in particular, of cholesterol homeostasis in the skin is of great importance. ABCA1 is a membrane transporter responsible for cholesterol efflux and plays a key role in maintaining cellular cholesterol levels. Among the many factors that have been associated with skin diseases, the environmental stressor cigarette smoke has been recently studied. In the present study, we demonstrate that ABCA1 expression in human cells (HaCaT) was increased (both mRNA and protein levels) after CS exposure. This effect was mediated by the inhibition of NFkB (aldehydes adducts formation) that allows the translocation of liver X receptor (LXR). These findings suggest that passive smoking may play a role in skin cholesterol levels and thus affect cutaneous tissues functions.

## 1. Introduction

Cutaneous tissue is the first barrier between the organism and the environment, preventing water and solute loss and defending us from chemical and physical assaults. The upper layer of the skin is the stratum corneum (SC) and consists of protein-enriched cells (corneocytes with cornified envelope) and lipid-enriched intercellular domains. During epidermal differentiation, the lipids are synthesized by the keratinocytes and are then extruded into the extracellular domains, where they form lipid-enriched extracellular layers [1]. Therefore, the synthesis and the trafficking of lipids and cholesterol in the skin are of extreme importance for this organ. Modifications in the lipid composition can lead to a disturbed skin barrier, which is an important component in the pathogenesis of contact dermatitis, ichthyosis, psoriasis, and atopic dermatitis [2].

Cholesterol levels are tightly regulated in keratinocytes/epidermis, and the maintenance of its homeostasis is of extreme importance for the skin physiology. In addition to being an essential component of all cell membranes, cholesterol is also required in keratinocytes to form lamellar bodies [3]. Cholesterol is also implicated in corneocyte desquamation and cohesion and keratinocyte differentiation [4]. Cholesterol homeostasis results from the balance between the *de novo* synthesis of cholesterol, the uptake of cholesterol from lipoproteins, and cholesterol efflux. The efflux of cholesterol from cells is mediated predominantly by ABCA1, which is a member of the ATP binding cassette transporter superfamily [5]. ABCA1 promotes the transport of cholesterol and phospholipids across cell membranes, where these lipids then complex with lipoproteins [6]. In addition, the activation of liver X receptor (LXR), a well known orphan receptor, increases ABCA1 gene expression [7].

Cutaneous tissue is one of the main organs of our body directly exposed to environmental stressors of which cigarette smoke (CS) is one of the most toxic. In the last 10 years, it has been shown that CS and the oxidative compounds that derive from the combustion of the cigarette can affect the skin [8,9]. Furthermore, several skin diseases such as melanoma, psoriasis and dermatitis have been now associated with CS exposure. CS contains thousands of chemical components, and its toxic effect is mainly due to the presence of free radicals compounds (ROS) and volatile electrophilic compounds such as α,β-unsaturated aldehydes. Acrolein (ACR) and 4-hydroxynonenal (4HNE) are the most reactive and toxic α,β-unsaturated aldehydes and they can be also generated during inflammation as a consequence of lipid peroxidation [10,11]. These aldehydes have been shown to be capable of affecting a variety of biochemical processes, including transcription factor activation and gene expression, production of inflammatory cytokines, respiratory burst activation, and cell death [12]. Recently, our group was able to demonstrate that CS exposure modulates genes involved in cholesterol trafficking such as SRB1 and ABCA1 in lung tissue [13].

As a continuation of our previous work, in this study we explored the modulation of ABCA1 in keratinocytes after CS exposure. Our results show that CS increased ABCA1 levels in keratinocytes by facilitating the translocation of LXR. This effect is a consequence of the inhibition of the transcription factor NFκB which form aldehydes-adducts.

## 2. Results and Discussion

### 2.1. The Effect of CS on ABCA1 Levels

**Figure 1:**
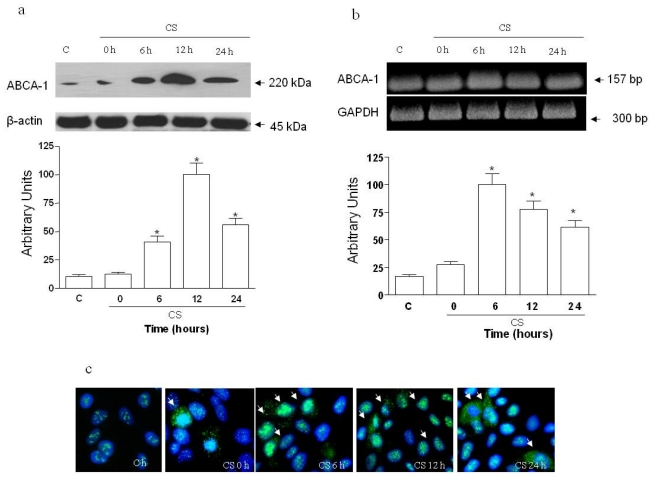
Exposure to CS increased ABCA1 protein (**a**) and mRNA (**b**) expression in HaCaT cells. Cells were exposed to CS for 50 min and cells were harvested at different time points (0–24 h). CS 0 h means cells harvested immediately after CS exposure (50 min). Quantification of the ABCA1 bands is shown in the bottom panel (averages of five different experiments, *p < 0.05). ICC for ABCA1 is shown in (**c**) (see arrows).

We first assessed whether ABCA1 is regulated by CS exposure in HaCaT cells. As shown in Figure 1a, ABCA1 protein levels increased markedly starting at 6 h (4-fold) and reaching an almost 9-fold increase 12 h after CS exposure. The induction of ABCA1 was also noted at the mRNA level (Figure 1b), where the most evident increase was 6 h after the exposure and was then reduced in the following time points (12 and 24 h). The increased levels of ABCA1 protein was also confirmed by immunocytochemistry (ICC) as shown in Figure 1c.

### 2.2 CS Induces LXR Activation

**Figure 2:**
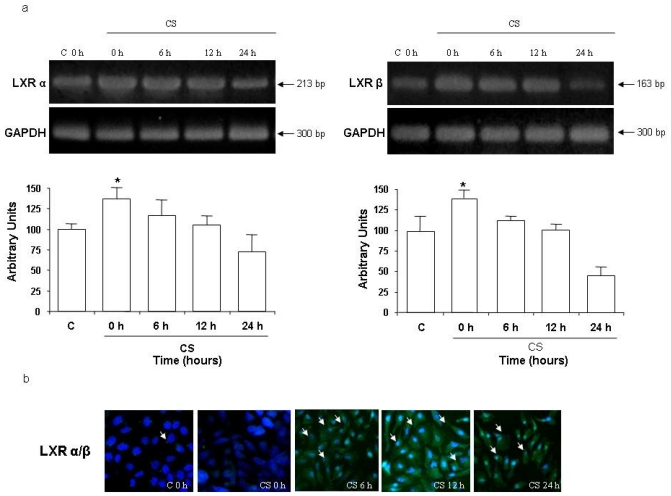
Exposure to CS increased LXRα and LXRβ mRNA in HaCaT cells. Cells were exposed to CS for 50 min and were harvested at different time points (0–24 h). CS 0 h means cells harvested immediately after CS exposure (50 min). Quantification of the LXRα and LXRβ bands is shown at the bottom panel (averages of five different experiments, *p < 0.05) (**a**). ICC for LXRα/β (marked by arrows) (**b**).

We next investigated whether the induction of ABCA1 was through the activation of LXR. As shown in Figure 2a, LXRα and LXRβ mRNA increased significantly after 6 h of CS exposure (*circa* 15%) and returned to the steady levels at 24 h. The activation of LXR induces its translocation, therefore we determined LXR translocation by ICC analysis. As shown in Figure 2b, 12 h after CS exposure there was an evident LXR translocation (arrows).

### 2.3. CS Induces NFκB-Aldehydes Adducts

**Figure 3:**
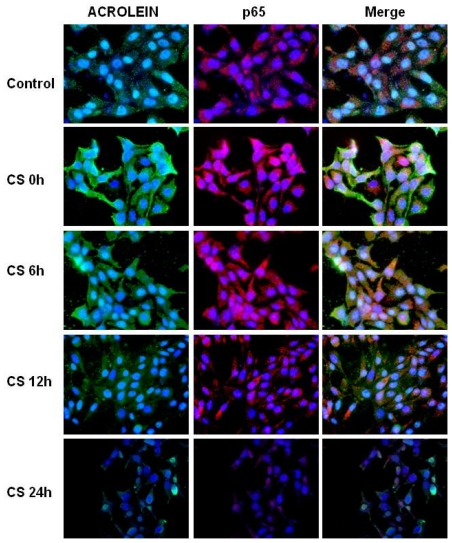
CS induces the increase of ACR-p65 adducts. Immunocytochemistry of HaCaT cells showing localization of ACR-adducts (left column, green color), p65 (central column, red color) and ACR-p65 adducts (right column, yellow color) before, and several time points after, CS exposure. CS 0 h means cells immediately after CS exposure (50 min). Images are merged in the right panel and the yellow color indicates overlap of the staining.

Since LXR is also under the negative control of NFκB, we next determined the levels of acrolein and 4HNE in the cells after CS exposure and asked whether they affect NFκB activation as measured by its nuclear traslocation by ICC analysis. As shown in Figure 3, after CS exposure the levels of ACR increased dramatically and this effect was noted during the first 12 h.

**Figure 4:**
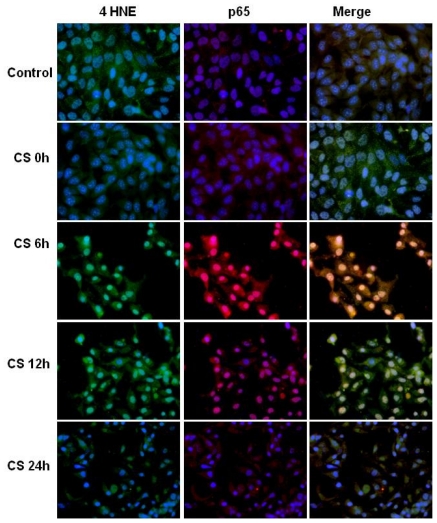
CS induced the increase of 4HNE-p65 adducts. Immunocytochemistry of HaCaT cells showing localization of 4HNE-adducts (left column, green color), p65 (central column, red color) and 4HNE-p65 adducts (right column, yellow color) before, and several time points after CS exposure. CS 0 h means cells immediately after CS exposure (50 min). Images are merged in the right panel and yellow color indicates overlap of the staining.

As shown in Figure 4 the same trend was also noted for 4HNE, although less dramatic and little time delayed (6 h).

In addition, after CS exposure the levels of NFκB increased (Figure 4 central column) and because LXR activation is partially regulated by NFκB inhibition, these data seem in disagreement with LXR activation. The results in the right column explain the disagreement since the merge column shows that there is co-localization (yellow). These data led us to support the idea that CS induces a direct covalent modification of NFκB mainly via protein adducts, mainly by 4HNE protein adducts.

**Figure 5:**
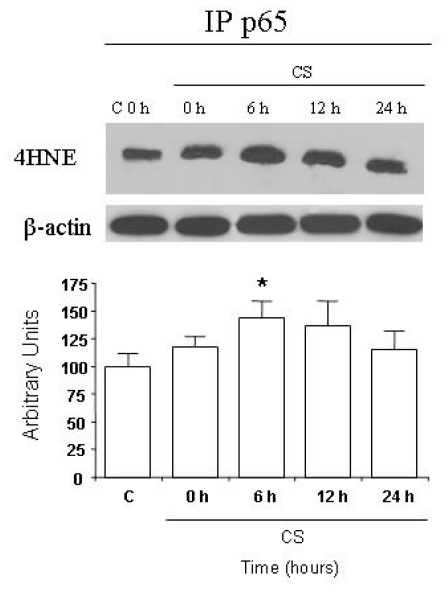
HaCaT cells were exposed to CS and cell lysates were immunoprecipitated using anti p65. Immunoprecipitated proteins were separated by SDS-PAGE, and then transferred to a nitrocellulose membrane and immunoblotted with anti-4HNE. Quantification of the 4HNE-p65 bands is shown in the bottom panel. The signals of the protein levels were determined by densitometric analysis of the scanned images. CS 0 h means cells harvested immediately after CS exposure (50 min). All samples were normalized per protein per sample. Data are expressed in arbitrary units. The Western blot shown at the top is representative of five experiments (*p < 0.05).

To confirm this result, we employed antibodies against 4HNE–protein conjugates in combination with p65 antibody in reciprocal immunoprecipitation–Western blot analyses. As shown in Figure 5, the IP experiments show the interaction between p65 and 4HNE, although not dramatic, was still significant.

One environmental factor that is known to be a major risk for disease is cigarette smoke exposure. Cigarette smoke is a complex chemical mixture containing thousands of different compounds; many of them are known to be carcinogens, co-carcinogens, mutagens or tumor promoters [17]. In recent years, there has been special emphasis placed upon the relevance of environmental or passive cigarette smoke and skin disease, also because recently it has been reported that sidestream smoke is more toxic than mainstream smoke, based on its chemical composition [18]. Environmental cigarette smoke contains not only a large amount of oxygen radical forming substances, but also very reactive aldehydes such as acrolein and 4HNE, which are known to disturb biological systems like the skin by reacting with a variety of their constitute molecules [19,20].

CS has not only been associated with lung pathologies but it has been shown to lead to many dermatological conditions, including poor wound healing, premature skin aging, squamous cell carcinoma, melanoma, oral cancer, acne, psoriasis, eczema, and hair loss [21].

Our study shows that CS exposure increased the levels of the cholesterol transporter ABCA1 and this might influence skin permeability that depends on the extracellular lipid contents located in the SC. Cholesterol represent *circa* one-quarter of the lipid contents of the SC and a variation of its transporter can lead to a modification of skin lipid composition and therefore alteration of skin permeability.

Expression of ABCA1 can be upregulated by liver X receptor (LXR) [22]. After activation, LXR forms a heterodimer with another nuclear protein, retinoid X receptor. The heterodimer of LXR/retinoid X receptor binds to the LXR response element (LXRE) in the proximal region of the ABCA1 gene promoter and induces ABCA1 transcription. Our data agrees with this pathway since we found a clear induction of LXR mRNA and also an increase of its translocation after CS exposure.

It has been reported that downregulation of ABCA1 is observed following exposure to inflammatory stimuli including IL-1β, TNF-α, IFN-γ, and LPS [23], in which the LXR-dependent or -independent pathway and transcriptional/posttranscriptional level regulation were reported to be involved. A recent study demonstrates a novel transcription mechanism through which IL-1beta downregulates ABCA1 by ROS-mediated upregulation of NFκB but not LXR in human monocytic leukemia cell line and lung cells. In our study, instead we can suppose that the upregulation of ABCA1 by CS could be mediated by LXR since the mRNA of this nuclear receptor increased after CS exposure.

Because many of the toxic effect of CS can be linked to the production of aldehydes, we determine the levels of acrolein and 4HNE in keratinocytes exposed to CS. As expected, the levels of the two aldehydes in the cells clearly increased.

NFκB is also a critical regulator involved in nuclear receptor signaling like LXR [24]. We investigated whether NFκB was involved in the upregulation of LXR, which can explain the increased levels of ABCA1 induced by CS.

Many studies indicate that acrolein and 4HNE are highly toxic compounds and they can be both present in tobacco smoke and can also be generated endogenously during lipid peroxidation. Because of their electrophile nature, they are able to form adducts with proteins, especially side chains of cysteine, histidine and lysine residues as well as free N-terminal residues. Our data shows that both acrolein and 4HNE are able to form adducts with NFκB and this could affect its ability to bind DNA after translocation.

It has been already reported that 4HNE and acrolein are able to suppress NFκB activation in different cell types although the exact mechanism is not clear yet [25]. The present results do not establish the precise mechanism involved in the inhibition of NFκB activation by CS, but we can suggest an interference with downstream events involved in NFκB activation. It has also been suggested that an upstream event leads to NFκB inhibition for both acrolein and 4HNE [26]. This can be a consequence of a direct modification of IKKβ by a direct reaction with a cystein residue in the activation loop of this protein [27].

## 3. Experimental Section

### 3.1. Cell Culture and Treatments

The HaCaT cell line (gift from Dr. F. Virgili ) were grown in Dulbecco’s modified Eagle’s medium High Glucose (Lonza, Milan, Italy), supplemented with 10% FBS, 100 U/mL penicillin, 100 μg/mL streptomycin and 2 mM l-glutamine as previously described [14]. Cell suspension containing 10 or 1 × 10^5^ viable cells/mL were used. Cells were incubated at 37 °C for 24 h in 95% air/5% CO_2_ until 80% confluency.

HaCaT cells were treated with either ACR (Aldrich, Milwaukee, WI) or 4HNE (Calbiochem, La Jolla, CA) for 30 min, and resuspended in serum-free DMEM medium (Lonza, Milan, Italy) for several time points. Stock solutions of ACR or 4HNE were made in sterile phosphate buffered saline (PBS) immediately before use. After treatments for various time periods, cells were collected by centrifugation for the several assays described below.

### 3.2. CS Exposure

Prior to CS exposure, the cells media was aspirated, and cells were placed in fresh serum-free medium for 50 min during CS exposure. Control cells were exposed to filtered air for the same duration (50 min).

The time and the method of exposure were chosen based on our previous work [13–15]. Under our experimental conditions no differences in cell viability as measured by Trypan blue exclusion was detected between control (air) and CS treated cells (data not shown).

HaCaT cells were exposed to fresh CS in an exposure system that generated CS by burning one UK research cigarette (12 mg tar, 1.1 mg nicotine) using a vacuum pump to draw air through the burning cigarette and leading the smoke stream over the cell cultures as described previously by our group [13]. After the exposure (air or CS), fresh media supplemented with 10% FBS was added to the cells.

### 3.3. Immunocytochemistry

HaCaT cells grown on coverslips at a density of 1 x 10^5^ cell/mL, were treated as indicated above and fixed in 4% paraformaldehyde in PBS for 30 min at 4°C. Cells were permeabilized for 15 min at RT with PBS containing 1% BSA, 0.2% Triton X-100, and 0.02% sodium azide, then the coverslips were blocked in PBS containing 1% BSA, 0.2% Nonidet P-40 and 0.02% sodium azide at RT for 1 h. Coverslips were then incubated for 1 h with primary antibody, followed by 1 h with secondary antibodies. Nuclei were stained with 1 μg/mL DAPI (Molecular Probes) for 1 min after removal of secondary antibodies. Coverslips were mounted onto glass slides using with anti-fade mounting medium 1,4 diazabicyclo octane in glycerine (DABCO) and examined by the Zeiss Axioplan2 light microscope equipped with epifluorescence at 40× magnification. Negative controls for the immunostaining experiments were performed by omitting primary antibodies. Images were acquired and analyzed with Axio Vision Release 4.6.3 software.

### 3.4. Western Blot Analysis

Total cell lysates were extracted in solubilization buffer containing 50 mM Tris (pH 7.5), 150 mM NaCl, 10% glycerol, 1% Nonidet P-40, 1 mM EGTA, 0.1% SDS, 5 mM N-ethylmaleamide (Sigma-Aldrich Corp.), protease and phosphatase inhibitor cocktails (Sigma–Aldrich Corp.) as described before [17].

Cells were harvested by centrifugation and protein concentration of the supernatant determined by the method of Bradford (Biorad Protein assay, Milan, Italy). Samples of 50–60 μg protein in 3× loading buffer (65 mM Tris base, pH 7.4, 20% glycerol, 2% sodium dodecyl sulfate, 5% β-mercaptoethanol and 1% bromophenol blue) were boiled for 5 min, loaded onto 10% sodium dodecyl sulphate–polyacrylamide electrophoresis gels and separated by molecular size. The gels were then electro-blotted onto nitrocellulose membranes which were then blocked for 1 h in Tris-buffered saline, pH 7.5, containing 0.5% Tween 20 and 5% milk. Membranes were incubated overnight at 4 °C with the appropriate primary antibody: ABCA-1, beta-actin (Abcam, Cambridge, MA), 4HNE (Millipore Corporation, Billerica, MA, USA) and acrolein (gift Prof. Ucida). The membranes were then incubated with horseradish peroxidase-conjugated secondary antibody (Sigma-Aldrich, Milan, Italy) for 1 h, and the bound antibodies were detected using chemiluminescence (BioRad, Milan, Italy).The blots were then stripped and re-probed with beta-actin (1:1000) as loading control. Images of the bands were digitized and the densitometry of the bands were performed using NIH-Image software.

### 3.5. Reverse Transcriptase-Polymerase Chain Reaction (RT-PCR)

Total RNA was extracted from HaCaT using RNAeasy mini kit (Qiagen, Hilden, Germany) and resuspended in 30 μL RNAse-free water. RNA integrity was checked on a 1.6% agarose gel (Amersham Pharmacia Biotech). Reverse transcription was performed using 0.5 μg of total pure RNA, 100 ng of antisense specific primer (MWG-Biotech/M-Medical, Milano, Italy), 100 units M-MLV reverse transcriptase (Promega, Madison, WI, USA), 20 units RNasin (Promega), 800 μM PCR Nucleotide Mix (Promega) as described by the manufacturers.

The cDNA were amplified by PCR using the specific sense and antisense primers (see Table 1), GoTaq Green Master Mix 2X (Promega), 5 μL RT-product, in a final volume of 50 μL. Amplifications were performed in a thermal cycler (PCR Sprint, Hybaid, UK), under the following conditions: 35 cycles at 93 °C 30 s, 58 °C 45 s, 72 °C 1 min for ABCA-1; 94 °C 5 min; 30 cycles at 93 °C 30 s, 58 °C 45 s, 72 °C 1 min, for LXRα and β; 25 cycles at 93 °C 30 s, 56 °C 45 s, 72 °C 1 min, for GAPDH. Negative control was performed without reverse transcriptase.

mRNA expression of the gene of interested was normalized by using GAPDH mRNA expression levels. The mRNA level analysis was done in triplicate and repeated.

### 3.6. Immunoprecipitation

Cell lysates containing 300 μg of protein were mixed with Dynabeads protein G and 2 μg of polyclonal antibody against NFκB (Santa Cruz). Following immunoprecipitation of NFκB, the presence of 4HNE adducts was determined, after which proteins were separated by SDS-PAGE, electrotransferred to nitrocellulose membranes, and immunoblotted with a 4HNE antibody (Millipore Corporation, Billerica, MA, USA).

### 3.7. Statistical Analysis

For each of the variables tested, two-way analysis of variance (ANOVA) was used. A significant effect was indicated by a *P*-value < 0.05. Data are expressed as mean ± S.D. of triplicate determinations obtained in 5 separate experiments.

## 4. Conclusions

In summary, cholesterol homeostasis is very important in keratinocytes because of the unique needs of keratinocytes for cholesterol. In this study, we demonstrate that a key transporter in cholesterol efflux, ABCA1, is upregulated by CS exposure via LXR activation and that this can then modify the permeability barrier of the skin, which is the initial step of many skin diseases. Our study confirms how passive smoking is not only noxious for the respiratory tract but also to the cutaneous tissues.

An understanding of the effects of smoking on skin is relevant since it provides more tools to show the toxicity of CS. In addition, it can give patients who are more concerned about their appearance further motivation to stop smoking.

## Figures and Tables

**Table 1:** Selected RT-PCR primer sequences and product size.

Target sequence	Primer	Sequence (5′→3′)	Amplicon length
ABCA-1	Sense	AACAGTTTGTGGCCCTTTTG	157 bp
	Antisense	AGTTCCAGGCTGGGGTACTT	
LXRα	Sense	CGGGCTTCCACTACAATGTT	213 bp
	Antisense	TCAGGCGGATCTGTTCTTCT	
LXRβ	Sense	CCTCCTGAAGGCATCCACTA	163 bp
	Antisense	GAACTCGAAGATGGGGTTGA	
GAPDH	Sense	ATGGGGAAGGTGAAGGTCGG	300 bp
	Antisense	TGGTGAAGACGCCAGTGGAC	
